# Pleurodesis by erythromycin, tetracycline, Aerosil™ 200, and erythromycin plus Aerosil™ 200 in a rat model: a preliminary study

**DOI:** 10.1186/2008-2231-20-79

**Published:** 2012-11-26

**Authors:** Shahryar Hashemzadeh, Khosrow Hashemzadeh, Kamran Mamaghani, Elnaz Ansari, Raheleh Aligholipour, Samad EJ Golzari, Kamyar Ghabili

**Affiliations:** 1 Tuberculosis and Lung Disease Research Center, Tabriz University of Medical Sciences, Tabriz, Iran; 2 Department of Cardiovascular Surgery, Shahid Madani Hospital, Tabriz University of Medical Sciences, Tabriz, Iran; 3 Department of General and Thoracic Surgery, Tabriz Branch, Islamic Azad University, Tabriz, Iran; 4 Cardiovascular Research Center, Tabriz University of Medical Sciences, Tabriz, Iran; 5 Physical Medicine and Rehabilitation Research Center, Tabriz University of Medical Sciences, Tabriz, Iran

**Keywords:** Aerosil™ 200, Erythromycin, Pleurodesis, Silicon dioxide, Tetracycline

## Abstract

**Background:**

None of the current pleurodesing agents fulfil all the criteria for best pleural sclerosant. Therefore, the search for the ideal agent for chemical pleurodesis still continues. The aim of the present study was to compare the effectiveness of erythromycin, tetracycline, Aerosil™ 200 (hydrophilic fumed amorphous silica), and erythromycin plus Aerosil™ 200 in producing pleurodesis in rats. In the present study, talc was not used as a pleurodesing agent due to an unavailability of its sterile and pure form in Iran.

**Methods:**

Overall, 75 adult male Spraque-Dawley rats were randomized to 5 treatment groups. Each group received an intrapleural injection via 5 Fr Silastic tubes of one of the following sterile agents: 35mg/kg erythromycin in 2 ml of saline, 35mg/kg tetracycline in 2 ml of saline, 35mg/kg Aerosil™ 200 in 2ml of saline, erythromycin (35mg/kg in 2 ml of saline) plus Aerosil™ 200 (35mg/kg in 2 ml of saline), or 2 ml of saline as a control. The animals were euthanized and necropsied 30 days after injection. The pleurae were assessed for macroscopic and microscopic evidence of surrounding inflammation and fibrosis.

**Results:**

The median macroscopic score in the Aerosil™ 200 group was significantly higher than that in the erythromycin group (*P* < 0.005). The median microscopic score in the erythromycin group was significantly lower than that in the Aerosil™ 200 and erythromycin plus Aerosil™ 200 groups (*P* < 0.005). Furthermore, maximum and minimum pleural fibrosis was observed in the erythromycin plus Aerosil™ 200 and erythromycin groups, respectively (*P* < 0.05).

**Conclusion:**

This study suggests that Aerosil™ 200 with or without erythromycin may be more potent pleurodesis agent than erythromycin and tetracycline.

## Background

Obliteration of the pleural space (pleurodesis) to prevent recurrent pleural effusion (mostly malignant) or pneumothorax is chiefly achieved through the use of chemical pleural sclerosants [1]. The best pleural sclerosant should be safe, inexpensive, widely available, and easily administered. However, none of the pleurodesing agents including talc, the sclerosant of choice in clinical practice, fulfil all these criteria [1-3]. Intrapleural administration of the most preferred pleurodesing agent, the talc, is believed to accompany with severe complications such as adult respiratory distress syndrome. On the other hand, parenteral tetracycline which was once the sclerosant agent of choice in clinical practice is no longer commercially available [4]. In addition, intrapleural application of some antineoplastic agents such as bleomycin is costly [5]. Therefore, the search for the ideal agent for chemical pleurodesis still continues.

Silica is the common name for silicon dioxide (SiO_2_). Silica may have a crystalline or a non-crystalline (amorphous) structure. Among synthetic amorphous silicas, pyrogenic (fumed) silica is widely used as reinforcing filler for silicon rubber, thickening and anti-settling agents in liquid systems of coating, adhesives, printing inks and cosmetics [6]. Aerosil™ 200, a hydrophilic fumed silica, is the most widely used form of the amorphous silica. Silica-based materials have been of great interest in chemical pleurodesis. Bioglass, a silica-based biomaterial similar to the Aerosil™ 200, has been found as a pleurodesing agent as effective as talc [7,8]. The pleurodesing effect of bioglass, talc and Aerosil™ 200 may be attributed to their major content, the silicon dioxide. Interestingly, pulmonary changes following inhalation of Aerosil™ 200 have been reported in the literature [9,10]. However, pleurodesing effect of Aerosil™ 200 has not been hitherto investigated. Therefore, the aim of the present study was to compare the effectiveness of erythromycin, tetracycline, Aerosil™ 200, and erythromycin plus Aerosil™ 200 in producing pleurodesis in rats compared with saline control. We did not investigate talc as a sclerosing agent in the present study due to an unavailability of its sterile and pure form.

## Methods

Adult male Spraque-Dawley rats were used in this study in accordance with the NIH Guide for the Care and Use of Laboratory Animals and local institutional guidelines for humane use of animals in research. The animals were housed three to five per cage and were provided with free access to compact food and water. Their food consisted of all essential ingredients including vitamins and minerals. The animals were kept under constant laboratory conditions with respect to humidity, illumination and temperature before and after surgery [11,12].

Overall, 75 adult male Spraque-Dawley rats, weighing 220–250 g, were anesthetized with an intramuscular injection of xylazine hydrochloride (5mg/kg) and ketamine hydrochloride (35mg/kg). Under the sterile conditions, a 1-cm incision was made over the seventh rib in the posterior axillary line on the rat’s right side, and a subcutaneous tunnel was extended to the fifth intercostal space. A 5 Fr Silastic tube was inserted through the incision and placed in the right pleural cavity. The rats were randomly assigned to one of five intrapleural treatment groups as follows: fifteen rats received erythromycin ethylsuccinate (35mg/kg) in 2 ml of saline (0.9% NaCl); fifteen animals were given tetracycline (35mg/kg) in 2 ml of saline; fifteen rats were given sterile Aerosil™ 200 (35mg/kg) in 2 ml of saline; fifteen animals received erythromycin ethylsuccinate (35mg/kg in 2 ml of saline) plus sterile Aerosil™ 200 (35mg/kg in 2 ml of saline); and fifteen control animals received 2 ml of normal saline. All the sclerosing agents were given sterile. These agents were instilled into the tube and flushed with air, and the animals were rotated to assure dispersion to the entire pleural surface. The control group received normal saline in a similar approach. All residual air was aspirated from the pleural space. After a perioperative chest radiograph demonstrated a fully expanded lung, the tube was removed, and the incision was closed. All animals were left to heal with both lungs expanded.

All animals in the experimental and control groups were euthanized on 30^th^ postoperative day by intravenous Beuthanasia-D, a pentobarbital/phenytoin commercial euthanasia solution. The thorax was removed en bloc. Lungs were fully expanded by instilling 10% formalin into trachea. After tracheal injection, the entire thorax was submerged in 10% neutral buffered formalin for 48 hours. Thereafter, a veterinary pathologist, blinded to the treatment group, performed thoracic necropsy. On macroscopic and gross observations, pleurodesis was graded as: 0 = normal pleura, 1 = multifocal fibrosis, 2 = diffuse adhesions and 3 = complete obliteration [2,13]. For microscopic evaluation, tissue sections from the lung and pleura were prepared and stained with hematoxylin and eosin (HE) and Masson’s trichrome. Light microscopic assessment of the sections was performed by a pathologist blinded to the study protocol. The slides were graded for inflammation/cellularity and scored using the following system: 0 (absence of cellularity and neovascularisation), 1 (mild cellularity and neovascularisation), 2 (moderate cellularity and neovascularisation), and 3 (severe cellularity and neovascularisation) [14]. Moreover, microscopic fibrosis was defined based on the similar grading system as absent (0), mild (1), moderate (2), and severe (3).

Data were presented as mean = standard deviation (SD), median or percentage. Statistical analysis was performed with SPSS for windows version 13.0. The chi-square test (*P* < 0.05) was used to compare the incidence of pleural fibrosis, pleural cellularity and neovascularization, and pleural adhesions among the groups. The Kruskal-Wallis test (*P* < 0.05) was used to compare the median macroscopic, microscopic, cellularity, neovascularization and fibrosis scores among the groups. Thereafter, comparisons between two groups were conducted with the Mann–Whitney ranking test. In order to avoid accumulation of errors due to multiple comparisons, the significance level was modified dividing it (*P* < 0.05) by the number of comparisons made (Bonferroni correction) with *P* < 0.005 considered as significant.

## Results

### Gross necropsy

**Figure 1:**
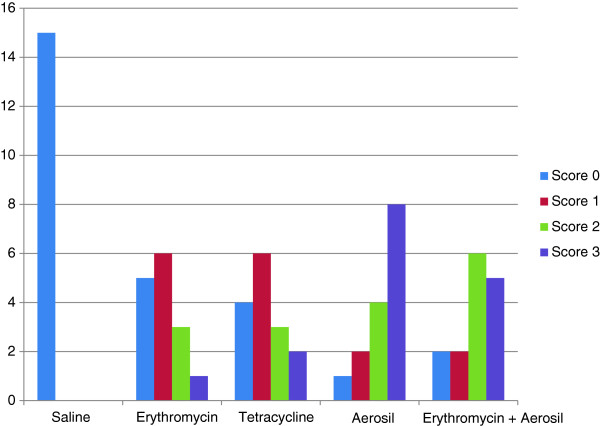
**Frequency of macroscopic scores in the treatment groups. **Microscopic scores were 0 = normal pleura, 1 = multifocal fibrosis, 2 = diffuse adhesions and 3 = complete obliteration.

No deaths occurred in any of the studied groups. At 30 days, the normal saline (control) group produced no considerable adhesions or pleurodesis on gross observation. In the erythromycin group, adhesions were grossly visible between the parietal and the visceral pleurae in 66.6% (10/15) of the rats. Although Aerosil™ 200 group had the greatest number of rats with pleural adhesions (93.3%), there was no significant difference between the studied groups in this regard (*P* > 0.05, Table 1). Furthermore, Aerosil™ 200 and erythromycin plus Aerosil™ 200 groups showed higher numbers of diffuse adhesion (score 2) and complete obliteration (score 3) on gross observation (*P* > 0.05, Figure 1). The median macroscopic score in the Aerosil™ 200 group was significantly higher than that in the erythromycin group (*P* < 0.005, Bonferroni-corrected Mann–Whitney *U* test, Table 2).

**Table 1:** Treatment-related microscopic and macroscopic findings, n (%)

	***Saline (n = 15)***	***Erythromycin (n = 15)***	***Tetracycline (n = 15)***	***Aerosil™ 200 (n = 15)***	***Erythromycin + Aerosil™ 200 (n = 15)***
Pleural fibrosis	0 (0)	6 (40%)^*^	11 (73.3%)	11 (73.3%)	14 (93.3%)^**^
Pleural inflammation	0 (0)	15 (100%)	15 (100%)	15 (100%)	15 (100%)
Pleural adhesions	0 (0)	10 (66.6%)	11 (73.3%)	14 (93.3%)	13 (86.6%)

Erythromycin treatment and erythromycin plus Aerosil™ 200 treatment significantly resulted in the minimum and maximum number of the pleural fibrosis, respectively (^*^P = 0.007; ^**^P = 0.02, Chi-square test).

**Table 2:** Treatment-related macroscopic, microscopic, cellularity, neovascularization and fibrosis scores, mean ± standard deviation (median)

	***Saline***^***a***^***(n = 15)***	***Erythromycin***^***b***^***(n = 15)***	***Tetracycline***^***c***^***(n = 15)***	***Aerosil™ 200 (n = 15)***	***Erythromycin + Aerosil™ 200***^***d***^***(n = 15)***	***P value***
Macroscopic score	0 ± 0 (0)	1 ± 0.9 (1)	1.2 ± 1 (1)	2.3 ± 1 (3)	1.9 ± 1 (1)	<0.001
Microscopic score	0 ± 0 (0)	1.2 ± 0.4 (1)	1.5 ± 0.5 (1)	2.1 ± 0.7 (2)	2.1 ± 0.7 (2)	<0.001
Cellularity score	0 ± 0 (0)	1.2 ± 0.4 (1)	1.1 ± 0.4 (1)	2 ± 0.7 (2)	1.7 ± 0.9 (1)	<0.001
Neovascularization score	0 ± 0 (0)	0.8 ± 0.4 (1)	1.4 ± 0.5 (1)	1.1 ± 1.1 (1)	2.1 ± 0.8 (2)	<0.001
Fibrosis score	0 ± 0 (0)	0.4 ± 0.5 (0)	1.3 ± 0.9 (1)	1.5 ± 1.2 (1)	1.1 ± 0.5 (1)	<0.001

^a^ All other treatment groups significantly differed from the normal saline group (*P* < 0.001, Kruskal-Wallis test).

^b^ Macroscopic, microscopic and cellularity scores in erythromycin group were significantly different from Aerosil™ 200 group (*P* < 0.005, Bonferroni-corrected Mann–Whitney *U* test).

^c^ Cellularity score in tetracycline group was significantly different from Aerosil™ 200 group (*P* < 0.005, test as above).

^d^ Microscopic, neovascularization and fibrosis scores in erythromycin plus Aerosil™ 200 group were significantly different from the erythromycin group (*P* < 0.005, test as above).

### Histopathology

The normal saline (control) group produced no considerable cellularity, neovascularisation, or fibrosis on microscopic observation at 30 days. Microscopically, all the erythromycin-treated rats exhibited inflammation, while only 40% (6/15) had pleural fibrosis. Pleural fibrosis was significantly lower in the erythromycin group compared with other treatment groups (*P* = 0.007, Table 1). Moreover, all rats in the treatment groups showed pleural inflammation (Table 1). In addition, pleural fibrosis was significantly higher in the erythromycin plus Aerosil™ 200 group compared with other treatment groups (*P* = 0.02, Table 1).

**Figure 2:**
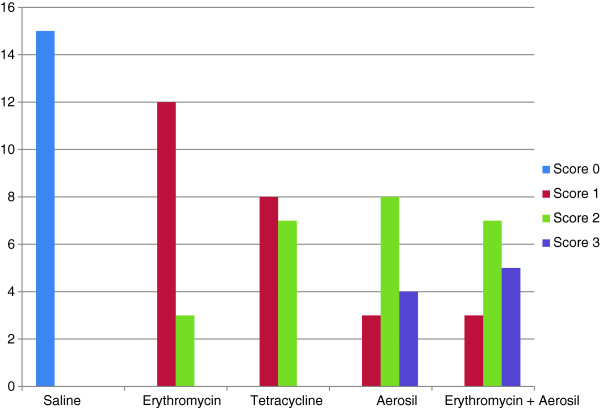
**Frequency of microscopic (inflammation/cellularity) scores in the treatment groups.** Microscopic (inflammation/cellularity) scores were 0 = absence of cellularity and neovascularisation, 1 = mild cellularity and neovascularisation, 2 = moderate cellularity and neovascularisation, and 3 = severe cellularity and neovascularisation.

Figure 2 illustrates the number of each microscopic (inflammation/cellularity) score in the studied treatment groups. Accordingly, none of the treatment groups had absence of cellularity and neovascularisation in the microscopic assessment (score 0). Furthermore, Aerosil™ 200 and erythromycin plus Aerosil™ 200 groups showed higher numbers of severe cellularity and neovascularisation (score 3) on microscopic observation (*P* > 0.05, Figure 2). Overall, the median microscopic score in the erythromycin group was significantly lower than that in the Aerosil™ 200 and erythromycin plus Aerosil™ 200 groups (*P* < 0.005, Bonferroni-corrected Mann–Whitney *U* test, Table 2). On the other hand, the Aerosil™ 200 group showed higher cellularity score than erythromycin and tetracycline groups (*P* < 0.005, Bonferroni-corrected Mann–Whitney *U* test, Table 2). In addition, neovascularization and fibrosis scores in erythromycin plus Aerosil™ 200 group were significantly higher than those in the erythromycin group (*P* < 0.005, Bonferroni-corrected Mann–Whitney *U* test, Table 2).

## Discussion

This rat model evaluated the effectiveness of erythromycin, tetracycline, Aerosil™ 200 and erythromycin plus Aerosil™ 200 as pleural sclerosing agents. This study demonstrated that Aerosil™ 200 with or without erythromycin was more potent pleurodesis agent both grossly and microscopically. To the best of our knowledge, the present study is the first investigation to study the pleurodesing effect of intrapleural Aerosil™ 200. However, there are a few studies on the health effects of Aerosil™ 200. In an animal investigation by Reuzel and colleagues, subchronic inhalation of Aerosil™ 200 induced the most severe changes in the lungs among other examined amorphous silicas [9]. In another study, Johnston et al. observed reversible inflammatory responses in bronchoalveolar lavage after inhalation of amorphous silica (Aerosil™ 200), while no mutagenic events were noted [10]. Likewise, recent experimental studies confirmed the pulmonary fibrogenetic effects of amorphous silica such as Aerosil™ 200 [15-17].

Chemical pleurodesis with silica-containing materials has been of utmost interest. Yeginsu and colleagues found that bioglass was as effective as talc in terms of pleurodesis [7,8]. It is believed that silicon dioxide or silica contributes to the pleurodesing effect of bioglass, talc and Aerosil™ 200. Silicon dioxide or silica exposure has been found to cause lung damage through direct cytotoxicity, generation of reactive oxygen species from alveolar macrophages, and stimulation of secretion of inflammatory cytokines, chemokines, and fibrogenic factors from alveolar macrophages and/or epithelial cells [7,18]. Although similar mechanism might be deemed for the Aerosil™ 200 particles, further investigations orchestrated on the mechanisms of Aerosil™ 200-induced pleurodesis are recommended.

Both in gross and microscopic assessments, erythromycin was less potent pleurodesis agent than the Aerosil™ 200. This finding is in contrast to that of the previous experimental study indicating that erythromycin was an ideal pleural sclerosing agent, compared with talc, doxycycline and diazepam [2]. Moreover, in a recent clinical study by Balassoulis et al., erythromycin was found as an effective and safe pleurodesing agent in patients with recurrent malignant pleural effusions [19]. However, we do not have an explanation for such a difference in the pleurodesing effect of erythromycin. Furthermore, tetracycline was not superior to the other pleurodesing agents in the present study. This finding is compatible with that of the previous reports; fibrin tissue adhesive [20], talc [21], and mitoxantrone were found as more effective sclerosing agents than tetracycline for pleurodesis [22]. In contrast, Vargas and colleagues found that tetracycline, rather than bleomycin and *Corynebacterium parvum*, was more potent pleurodesing agent in a rabbit model [23,24]. In a systematic review, tetracycline was reported to be associated with more recurrences when compared to talc [3]. The available clinical evidence supports the use of talc as the sclerosant of choice rather than other sclerosants (tetracycline, bleomycin, or mustine) [1].

This study has certain limitations. We did not investigate talc as a sclerosing agent in the present study. The rationale behind this exclusion was the lack of sterile and pure talc in Iran. Moreover, other routinely used pleurodesing agents such as bleomycin and parenteral doxycycline were not available due to their high costs. Therefore, further studies are recommended to be orchestrated in order to investigate the probable advantage of the Aerosil™ 200 over the most commonly used pleurodesis agents such as sterile and pure talc in terms of efficacy, safety, rapidity of action, mechanism of action or cost. In addition, one may consider the rabbit model as generally more established method for experimental pleurodesis. However, there are a number of experimental studies of pleurodesis on the rats [20,25-27]. Only single dose of 35 mg/kg was studied for each sclerosant, and different doses might have resulted in more favorable responses. However, the applied treatment dosages were based on those of the previous studies. Future studies with larger doses of erythromycin and tetracycline may achieve higher response rates. Furthermore, distant sequelae of the studied pleurodesing agents in the contralateral lung as well as the liver along with their systemic effects were not evaluated. Liver enzyme tests, histopathological assessment of the contralateral lung and the liver, and serum and tissue angiotensin-converting enzyme could have provided precious information regarding the toxicity of the studied pleurodesing agents, in particular the Aerosil™ 200. In addition, grading of the pleurodesis immediately after sacrificing the animals while the tissues were fresh and soft might have yielded to different results. The formalin exposure makes the tissues firm and shrunk, resulting in difficult evaluation of the pleural adhesions [8].

## Conclusion

In conclusion, this preliminary study suggests that Aerosil™ 200 with or without erythromycin may be more potent pleurodesis agent than erythromycin and tetracycline. As superiority between Aerosil™ 200 alone and Aerosil™ 200 plus erythromycin could not be determined with the results of the present study, further cost-effective investigations targeting at the distant sequelae of these treatments are required.

## Abbreviations

SiO2: Silicon dioxide; HE: Hematoxylin and eosin.

## Competing interests

The authors declare that they have no competing interests.

## Authors’ contributions

S.H., K.H., K.M., and R.A. participated in the design of study and performed the experimental parts of the research. E.A., S.G. and K.G. participated in acquisition of data, analysis and interpretation of data, drafting the article, and final approval of this version. All authors read and approved the final manuscript.
